# Haemorrhagic cystitis due to BK virus in a child with ALL on standard chemotherapy without stem cell transplant

**DOI:** 10.3332/ecancer.2013.350

**Published:** 2013-09-12

**Authors:** Samin Alavi, Mohammad Kaji Yazdi, Mahmoud Parvin, Farahnaz Zohrehbandian, Roxana Azma

**Affiliations:** 1 Pediatric Congenital Hematologic Disorders Research Center, Mofid Children’s Hospital, Shahid Beheshti University of Medical Sciences, Tehran 15468-15514, Iran; 2 Department of Pathology, Shahid Labbafinejad Medical Center, Shahid Beheshti University of Medical Sciences, Tehran 16666-63111, Iran; 3 Islamic Azad University, North branch, Tehran 16679-34783, Iran; 4 Radiology Department, Mofid Children’s Hospital, Shahid Beheshti University of Medical Sciences, Tehran 15468-15514, Iran

**Keywords:** BK virus, haemorrhagic cystitis, chemotherapy, acute leukaemia, nontransplantation

## Abstract

The BK virus (BKV) is a nonenveloped double-stranded DNA virus of the polyomavirus family that primarily affects immunocompromised people. BKV infects humans at an early age. Initial infections with BKV are mainly asymptomatic and usually remain latent in the brain, peripheral blood, kidneys, and urothelium. Following the primary infection, viruses persist indefinitely as ‘latent’ infections of the kidney and urinary system because the virus is urotheliotropic. Reactivation of the virus infections occurs in individuals with severe immunosuppression states such as kidney and stem cell transplantation and rarely in pregnancy. In this line, BKV has been implicated as a common cause of late-onset haemorrhagic cystitis (HC) in patients who have undergone stem cell transplantation. In contrast, reports of BKV-associated diseases in nontransplant paediatric patients are almost exclusively in patients with human immunodeficiency virus infection. Herein, we report the first case of a child with acute lymphoblastic leukaemia who developed BKV-associated HC without receiving stem cell transplantation while on standard maintenance chemotherapy.

## Introduction

Polyomavirus hominis 1, known as BK virus (BKV), infects up to 90% of the general population by adulthood. However, significant clinical manifestations are rare and restricted to individuals with impaired immune functions. BKV has been associated with various clinical entities such as haemorrhagic cystitis (HC), ureteric stenosis, vasculopathy, pneumonitis, encephalitis, retinitis, and even multiorgan failure [1]. BKV infection usually occurs during childhood at a median age of four to five years. The seroprevalence is lowest at the age of six months and increases by adulthood (range: 46–94%). Primary BKV infection is poorly characterised, most probably because of its subclinical or unspecific course. After the primary infection, BKV persists in the urinary tract as the principal site [2]. Two major diseases associated with BKV have been recognised: the polyomavirus-associated nephropathy in solid organ transplants (especially kidney) and HC in allogeneic haematopoietic stem cell transplantation (HSCT) recipients. BK-induced HC rarely follows solid organ transplantation. It is much more frequent following allogeneic HSCT (5–60%). Conversely, renal involvement is rare after allogeneic HSCT, but common among kidney transplant recipients [3]. Herein, we report the first case of a boy with acute lymphoblastic leukaemia (ALL) who developed severe haematuria and dysuria due to BKV while he was on standard maintenance chemotherapy without receiving any kind of stem cell transplantation.

## Case Report

**Figure 1.**
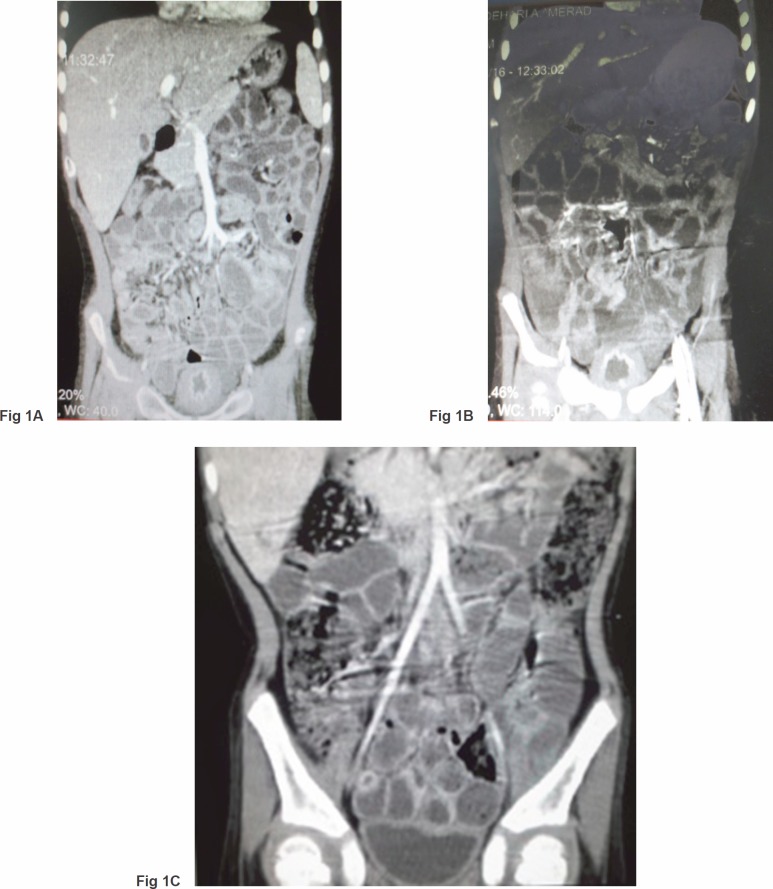
(A) Severe bladder wall thickness with distinct layering, perivesicular stranding, and reduced intravesical volume. (B) and (C) Remarkable improvement in bladder wall thickness and bladder space.

**Figure 2.**
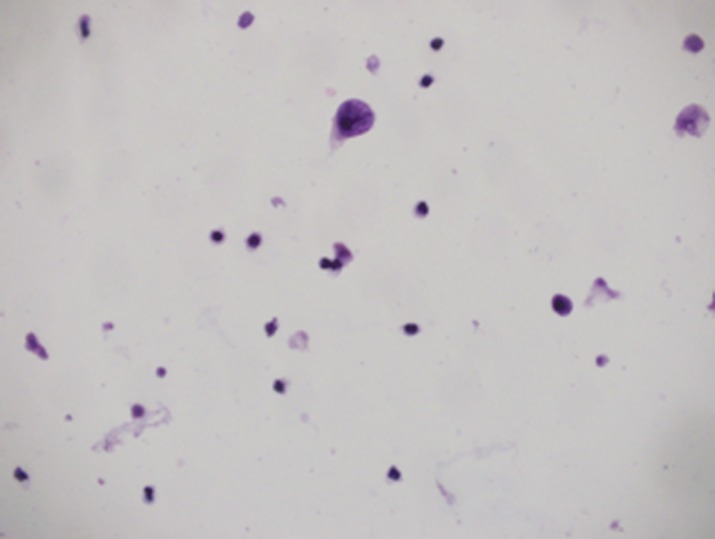
Decoy cell was found in the patient’s urine (detected by papanicolaou stain).

A five-year-old boy, a case of standard-risk ALL with *t*(12,21), developed a prolonged course of intermittent fever and productive cough at the end of the second year of maintenance chemotherapy. He was receiving chemotherapy protocol as ALL-BFM-90 with some modifications. Induction therapy consisted of vincristine, prednisone, doxorubicin, L-asparaginase (L-ASP) [(10,000 IU/m^2^), twice a week], and intrathecal chemotherapy, followed by consolidation therapy including cytarabine, L-Asp, etoposide, and intermediate dose methotrexate (1 g/m^2^) with leucovorin. Reinduction therapy was similar to induction therapy except that daunorubicin was substituted for doxorubicin and dexamethasone for prednisone, respectively. Cranial radiotherapy was not given to the patient. Continuation therapy consisted of 6-mercaptopurine and methotrexate along with vincristine and prednisone every three weeks. A thorough investigation was conducted for evaluation of the fever and cough. A bone marrow examination was compatible with remission; a chest computed tomography (CT) scan demonstrated diffuse patchy alveolar infiltrations. Investigation for fungal infections, including imaging studies and serum galactomannan assay, was negative, whereas cytomegalovirus pp65 assay on the sputum was indicated to be positive. Hence, he was determined to receive a course of ganciclovir along with the broad spectrum antibiotics including ceftazidime and vancomycin. The fever and cough improved significantly, and he was discharged after three weeks of treatment. Two days later, he came back with a severe dysuria and macroscopic haematuria. Aggressive hydration to ensure a urine output of 4–5 litres/day with 1/3 normal saline was started for the patient. His platelet counts and coagulation profile were normal. An imaging study of the urinary system, including a contrast enhanced abdominopelvic CT scan, showed a very thick walled bladder with distinct layering, perivesicular stranding, and intravesical clots [Figure 1(A)]. There was no recent history of treatment with cyclophosphamide in the patient. Accordingly, a urine sample was checked for BKV by real-time polymerase chain reaction (PCR), which was positive with increased loads to 1 × 10^5^ copies/ml. At the same time, urine was also checked by PCR for cytomegalovirus (CMV) and adenovirus, which were negative. In addition, complementary for diagnosis, urine was examined for observation of decoy cells that were positive on centrifuged spot urine sample of the patient (Figure 2). The patient’s plasma was also tested and showed positive viraemia with BKV with 3,000 copies/ml of plasma. The patient was so frustrated that urinary catheterisation was inserted for the child through which bladder irrigation was performed. He also received intravenous immunoglobulin (IVIG), intravenous ciprofloxacin, and a combination of urinary antispasmodics; oxybutynin and phenazopyridine as local urinary analgesic to help in providing immediate symptomatic relief. The symptoms started to subside slowly so that during 10–12 weeks of conventional therapy, a decrease in bladder wall thickness was observed on repeated CT scans [Figure 1(B) and (C)]. Likewise, urine BK viral load started to decline; however, as mild degrees of dysuria was persistent for almost two months, the child discharged with oral ciprofloxacin and topical application of lidocaine and a mild steroid cream for symptomatic relief.

## Discussion

BKV-associated HC is a well-known common complication in patients after kidney and bone marrow transplantation. In allogeneic SCT patients, BKV is the most significant cause of virus-associated HC; however, it is rare among other immunosuppressed patients. In contrast to reports in the bone marrow transplant literature where the disease is almost exclusively due to viral reactivation, almost one-quarter of BKV infections in kidney transplant recipients are primary infections [4]. This report demonstrates the first case of BKV-associated HC in a young child with ALL on standard chemotherapy. BK viraemia is remarkably associated with late-onset HC and cases of BK-HC have been shown to have a significantly higher peak of BK plasma and urine viral load than controls [5, 6]. In our case, there was an increased load of BKV-DNA with 1 × 10^5^ copies/ml in urine and also 3,000 copies/ml in plasma, which obviously decreased during the following weeks by reduction of immunosuppression and application of conventional treatments for the patient. Azzi *et al *reported three cases out of 55 adults with refractory or relapsed ALL who developed HC and BK viruria [7]; albeit, they had also received moderate doses of cyclophosphamide for seven days as continuous infusion without receiving Mesna prophylaxis as a urinary protection. As a matter of fact, in Azzi’s study, haematuria could not be attributed to BKV alone. In another study, four paediatric oncology patients developed HC after high-dose cyclophosphamide chemotherapy without receiving transplant, despite aggressive hydration, and mesna. The presence of BKV in three of these patients may have been a predisposing factor for the development of HC early after exposure to high-dose cyclophosphamide [8]. There is also a report of HC due to BKV reactivation in a 15-year-old girl treated for stage 4 Hodgkin’s disease. This patient had also received previous courses of cyclophosphamide and referred with HC two weeks after the last course of chemotherapy [9]. None of these cases existing in the literature that developed BKV-HC were receiving merely a standard chemotherapy for ALL.

The association of CMV infection with HC was described by Spach *et al *[10], and a trend towards a higher incidence of HC in patients with reactivation of CMV was reported in a group of patients receiving T-cell-depleted allografts [11]. Our patient developed a prolonged course of intermittent fever and cough for which CMV assay of the sputum was performed and according to the positive results, the patient was treated with ganciclovir. Indeed, we presumed that CMV infection and its treatment by ganciclovir have been acting more as predisposing factors by induction of severe immunosuppression rather than being considered as a culprit in causing HC. Bielorai *et al *have hypothesised that as in JC virus, CMV was responsible for the replication of the BKV and for the resultant HC [12]. The hallmark of BKV infection in the urinary system is the presence of so-called ‘decoy cells’ in the urine. These cells arise from renal tubules especially the distal segments, collecting ducts, and uroepithelium [13, 14]. In our patient, decoy cells were observed in acute phase, persisting during successive weeks. Decoy cells could be detected by papanicolaou stain on fixed urine that has been centrifuged. They are cells with an enlarged nucleus, which is occupied by a basophilic inclusion enclosed by chromatin that confers a ground glass appearance [13].

Treatment options for BKV infection are limited. Pain relief is always recommended besides specific therapies. In the case that the patient is a receiver of a transplant, the most important strategy is reduced immune suppression. Treatment modalities that have been used include quinolone antibiotics, intravenous immune globulin, cidofovir, and the immunomodulatory drug, leflunomide [15]. Cidofovir (HPMPC) is a cytosine derivative, which has broad-spectrum activity against many DNA viruses including CMV, adenoviruses, and polyomaviruses [16]. Successful treatments with systemic and intravesical cidofovir have been reported [17]. Leflunomide is an immunosuppressive drug with antiviral activity. Preliminary data suggest that leflunomide could be potentially effective for treating BKV-HC without significant toxicity [18]. The quinolone antibiotics have been shown to inhibit BKV replication. Leung *et al *have shown that ciprofloxacin recipients who excrete the medication in urine do express anti-BKV activities. Ciprofloxacin has inhibitory effects on replication of BKV and has been suggested for prevention and treatment of BK-HC in immunosuppressed individuals [19]. We commenced treating the patient with intravenous ciprofloxacin along with IVIG regardless of patient’s age. In our patient, it seemed to be efficacious because BK viruria detected by PCR decreased gradually during the course of the treatment, whether it was due to some degree of immune recovery or actual positive effects of ciprofloxacin needs to be proved in future studies.

## Conclusion

To the best of the authors’ knowledge, this is the first case of BKV-associated HC in a five-year-old boy with ALL while receiving standard maintenance chemotherapy. BKV-HC was supposed to follow a course of severe immunosuppression due to the treatment of CMV pneumonitis by ganciclovir. Increased viral loads of BKV in the urine and plasma, detected by PCR gradually declined after reduced immunosuppression and prolonged treatment with ciprofloxacin. BKV-HC should be considered even in the setting of nontransplant patients who go through conditions with severe comprising of the immune function. Nonspecific treatments such as ciprofloxacin should be started for oncologic patients who develop severe haematuria and dysuria before more specific treatments could be employed.

## Conflict of Interest

Authors declare no conflict of interest.
